# Mild cognitive impairment is associated with poor physical function but not bone structure or density in late adulthood: findings from the Hertfordshire cohort study

**DOI:** 10.1007/s11657-018-0455-3

**Published:** 2018-04-24

**Authors:** A. Patel, K. A. Jameson, M. H. Edwards, K. Ward, C. R. Gale, C. Cooper, Elaine M. Dennison

**Affiliations:** 1 University Hospital Southampton NHS Foundation Trust, Southampton, UK; 2 MRC Lifecourse Epidemiology Unit, Southampton General Hospital, University of Southampton, Southampton, SO16 6YD UK; 3 Centre for Cognitive Ageing and Cognitive Epidemiology, Department of Psychology, University of Edinburgh, Edinburgh, UK; 4 NIHR Musculoskeletal Biomedical Research Unit, Nuffield Department of Orthopaedics, Rheumatology and Musculoskeletal Sciences, University of Oxford, Oxford, UK; 5 Victoria University, Wellington, New Zealand

**Keywords:** Mild cognitive impairment, Physical performance, Bone parameters, Epidemiology

## Abstract

***Summary*:**

This study investigated the association between mild cognitive impairment (MCI) and physical function and bone health in older adults. MCI was associated with poor physical performance but not bone mineral density or bone microarchitecture.

**Purpose:**

Cross-sectional study to investigate the association between mild cognitive impairment (MCI) and physical performance, and bone health, in a community-dwelling cohort of older adults.

**Methods:**

Cognitive function of 222 men and 221 women (mean age 75.5 and 75.8 years in men and women, respectively) was assessed by the Strawbridge questionnaire and Mini Mental State Exam (MMSE). Participants underwent dual-energy X-ray absorptiometry (DXA), peripheral-quantitative computed tomography (pQCT) and high-resolution peripheral-quantitative computed tomography (HR-pQCT) scans to assess their bone density, strength and microarchitecture. Their physical function was assessed and a physical performance (PP) score was recorded.

**Results:**

In the study, 11.8% of women and 8.1% of men were cognitively impaired on the MMSE (score < 24). On the Strawbridge questionnaire, 24% of women were deemed cognitively impaired compared to 22.3% of men. Cognitive impairment on the Strawbridge questionnaire was associated with poorer physical performance score in men but not in women in the unadjusted analysis. MMSE < 24 was strongly associated with the risk of low physical performance in men (OR 12.9, 95% CI 1.67, 99.8, *p* = 0.01). Higher MMSE score was associated with better physical performance in both sexes. Poorer cognitive function, whether assessed by the Strawbridge questionnaire, or by MMSE score, was not associated with bone density, shape or microarchitecture, in either sex.

**Conclusion:**

MCI in older adults was associated with poor physical performance, but not bone density, shape or microarchitecture.

## Introduction

Mild cognitive impairment (MCI) is a heterogeneous state between normal ageing and early dementia [1]. The prevalence of MCI is rising as the population ages. It has been estimated to affect 19% of individuals aged 65 and over [2]. While cognitive decline is a well-documented clinical hallmark of MCI, changes in physical health are also apparent during the course of MCI and include increased physical fragility and reduced physical performance [3–6], resulting in individuals being at increased risk of injuries [7].

Emerging data suggest bone mineral density (BMD) is reduced in severely cognitively impaired individuals, compared to cognitively normal individuals of a similar age [8]. Although it has been suggested that there may be a link between cognitive impairment and BMD [9], little is known about bone loss rates in MCI subjects.

The majority of evidence to date has been derived from studies set in long-term care/nursing homes and hospitals [7, 10–12] where individuals are frail and severely cognitively impaired. The purpose of this study was to further investigate whether an association exists between MCI and both bone health and physical performance status in a well-established community-dwelling cohort of older adults. If MCI was associated with reduced bone density, this might be considered an important risk factor for fracture that could be addressed in treatment algorithms that included anti-osteoporosis therapy.

## Methods

The Hertfordshire Cohort Study (HCS) is a population-based UK cohort of older adults. Study design and recruitment have been described in detail previously [13]. In brief, we traced men and women born between 1931 and 1939 in Hertfordshire and who still lived there in 1998–2003. A nurse-administered questionnaire, which included details of socioeconomic status and dietary calcium intake, was conducted at this time. In 2011–2012, 443 participants consented to a home visit by a trained research nurse. At this visit, a nurse-administered questionnaire was again administered which included details of smoking status, alcohol consumption and physical activity (average minutes per day spent walking, cycling, gardening, playing sport and doing housework in the last 2 weeks). Height was measured to the nearest 0.1 cm and weight to the nearest 0.1 kg on a SECA floor scale (Chasmors Ltd., London, UK). Body mass index (BMI) was calculated as weight divided by square of height (kg/m^2^).

All assessments detailed below were performed in 2011/2012.

## Cognitive function assessment

Participants completed a Mini Mental State Examination (MMSE) [14] and Strawbridge questionnaire. The Strawbridge questionnaire consisted of 16 questions assessing four functional domains: physical, nutritive, cognitive and sensory. The cognitive domain of the questionnaire was scored positive when difficulty was reported often or very often for paying attention, finding the right word, remembering things or remembering where something was put [15].

## Physical performance assessment

Hand grip strength was measured using a Jamar handheld dynamometer [16]. Participants undertook three physical performance tasks: (1) gait speed [17], (2) repetitive sit-to-stand [18] and (3) modified Tinetti balance test [19]. A physical performance score was scored according to Guralnik et al. [20]. Participants were given scores of 0 to 4, with best performance scoring 4. The scores for three physical performance tasks were then summed. The maximum possible score was 12 and the minimum was 0. In keeping with previous work [20], those with a score equal to or lower than 9 were deemed as having low physical performance.

## Dual-energy X-ray absorptiometry (DXA)

A DXA scan was performed to measure BMC (g) and aBMD (g/cm^2^) at the non-dominant hip using a Lunar Prodigy Advanced Scanner, UK. Positioning for all scans was completed in accordance with the manufacturer’s instructions. The effective dose to the subject during each scan was 0.009 μSv.

## Peripheral quantitative computed tomography (pQCT)

Distal radial and tibial pQCT scans were performed on the non-dominant side except when it had previously fractured (Stratec 2000 instrument, voxel size 80 μm). A scout view was performed on the lower leg to place a reference from which measurement sites are determined. Two slices were taken in the radial scan (4 and 66%). Four slices were taken for the lower leg scan (4, 14, 38 and 66%). Measurements were taken from both the radius and tibia total bone areas (TBAs), trabecular bone mineral density (tBMD), cortical bone mineral density (cBMD) and cortical bone area (cBA). Short-term measurement precision error was expressed as a coefficient of variation, ranged from 0.88% (total tibial density, 4% slice) to 8.8% (total radial area, 66% slice), but was typically between 1 and 3%. These figures were obtained using images acquired from 20 volunteers who were part of the study and hence were representative of the study population. The effective dose to the subject during each scan was 0.01 μSv.

## High-resolution peripheral quantitative tomography (HR-pQCT)

Each participant had measurements of the non-dominant distal radius and distal tibia using HR-pQCT (XtremeCT, Scanco Medical AG, Switzerland) except when it had previously been fractured in which case the dominant side was scanned. A total of 110 slices were obtained which represented a volume of bone 9 mm in axial length with a nominal resolution (voxel size) of 82 μm. Antero-posterior 2D scout views were performed to determine the region to be scanned. All scans were performed in keeping with the manufacturer’s guidelines and as described by Boutroy et al. [21].

Image analysis was carried out using the standard manufacturer’s method which has been described in detail previously [22]. Analysis produced the following outcomes: total BMD (Tt.vBMD, g/cm^3^), trabecular BMD (Tb.vBMD, g/cm^3^), trabecular number (Tb.N, per mm), trabecular thickness (Tb.Th, μm), trabecular separation (Tb.Sp, μm), cortical area (Ct.area, mm^2^), cortical density (Ct.vBMD, g/cm^3^), cortical porosity (Ct.Po, %) and cortical thickness (Ct.Th, μm). Short-term precision values for cortical and trabecular BMD have been shown to range from 0.3 to 1.2 [23]. The effective dose to the subject during each scan was < 3 μSv.

## Ethical permission

Ethical permission for the study was granted by the East and North Hertfordshire Ethical Committees. All participants gave written informed consent.

## Exclusion criteria

Study participants who were taking drugs that are known to alter bone metabolism (e.g. bisphosphonates) were excluded from the study. However, women who were taking hormone replacement therapy (HRT) were allowed to participate.

### Statistical analysis

Variables were assessed for normality and transformed where necessary using the Fisher-Yates rank-based inverse normal transformation to create *z*-scores (FY *z*-scores). Descriptive statistics for continuous variables were expressed as mean and standard deviation (SD) or median and interquartile range (IQR). Categorical variables were expressed as frequency and percentage (%). Differences between men and women were assessed using Student’s *t* tests, Mann-Whitney tests or Pearson’s *χ*^2^ tests, as appropriate. Linear and logistic regression analyses were used to examine the associations between being cognitively frail on the Strawbridge Cognitive Domain and MMSE score and physical performance, and bone outcomes. The regression analyses were undertaken with and without adjustment for the following lifestyle confounders: age, BMI, social class, smoker status, alcohol consumption, physical activity and dietary calcium intake, and, in women only, years since menopause and HRT use. Statistical analyses were performed using STATA, version 14.

## Results

### Study participants and characteristics

The characteristics of the study participants are displayed in Table 1. The mean (SD) age of participants was 75.5 (2.5) and 75.8 (2.6) years in men and women, respectively. On average, men were taller (*p* < 0.001) and heavier (*p* < 0.001) than women but BMI did not differ significantly by sex.

**Table 1:** Baseline characteristics of study participants

Descriptives	Men		Women				*p* value
Characteristics	*N*	Mean	SD	*N*	Mean	SD	
Age (years)	222	75.5	2.5	221	75.8	2.6	0.280
Height (cm)	221	172.7	6.5	217	158.8	6.1	< 0.001
Weight (kg)	221	82.2	1.2	221	70.2	1.2	< 0.001
BMI (kg/m^2^)	221	27.9	3.9	217	28.4	5.1	0.252
	*N*	Median	IQR	*N*	Median	IQR	*p* value
Activity time in last 2 weeks (min/day)	205	176.4	105.0–270.0	210	199.6	135.0–282.9	0.089
Daily dietary calcium intake (mg)	222	1221	1021–1432	221	1105	939–1281	< 0.001
Alcohol consumption (units per week)	222	6.5	1.0–14.0	221	0.5	0.0–3.5	< 0.001
	Total *N*	*N*	%	Total *N*	*N*	%	*p* value
Smoker status	222			221			< 0.001
Never		85	38.3		142	64.3	
Ex		126	56.8		73	33.0	
Current		11	5.0		6	2.7	
Social class	211			221			0.520
I–IIINM		89	42.2		100	45.2	
IIIM–V		122	57.8		121	54.8	
Years since menopause				218			
< 20 years					23	10.6	
≥ 20 to < 25 years					53	24.3	
≥ 25 to < 30 years					45	20.6	
≥ 30 to < 35 years					24	11.0	
≥ 35					11	5.0	
Hysterectomy					62	28.4	
HRT use				213			
Never					116	54.5	
5 years or more ago					91	42.7	
Current					6	2.8	

### MMSE and Strawbridge questionnaire assessments

The median (IQR) MMSE score was 28 (25–29) for both men and women. In the study, 11.8% of women were cognitively impaired on the MMSE (score < 24), compared to 8.1% of men. On the Strawbridge questionnaire, 24% of women were deemed cognitively impaired compared to 22.3% of men.

### Associations between cognitive impairment and physical performance outcomes

Men had a greater mean maximum grip strength compared to women, 36.1 and 21.3 kg, respectively (*p* < 0.001). Gait speed was faster in men than in women: 0.78 vs 0.73 m/s (*p* = 0.006). The median time for the repetitive sit-to-stand test in men (15.8 s, IQR 13.6–18.8) was less than that for women (17.0 s, IQR 13.8–20.2), *p* = 0.05. Fifty-six (26.3%) women were unable to perform the Tinetti balance test for greater than 10 s, compared to 43 (20.1%) men (*p* = 0.13). One hundred and forty-one women (71.2%) had a physical performance score of 9 or less, compared to 116 men (56.9%) (*p* = 0.003).

Table 2 shows the associations between being cognitively impaired as defined by the Strawbridge questionnaire and physical performance outcomes in men and women.

**Table 2:** Cognitive impairment as defined by the Strawbridge questionnaire as an explanatory variable for physical performance outcomes

	Men								Women							
	Unadjusted				Adjusted^a^				Unadjusted				Adjusted^a^			
	*N*	Regression coefficient	95% CI	*p* value	*N*	Regression coefficient	95% CI	*p* value	*N*	Regression coefficient	95% CI	*p* value	*N*	Regression coefficient	95% CI	*p* value
Grip (kg)	219	− 1.10	(− 3.46, 1.26)	0.361	191	0.12	(− 2.15, 2.39)	0.919	221	− 2.04	(− 3.87, − 0.21)	0.029	196	− 1.53	(− 3.50, 0.45)	0.130
Gait speed (FY *z*-score)	205	− 0.28	(− 0.62, 0.05)	0.094	178	− 0.21	(− 0.55, 0.12)	0.216	205	− 0.25	(− 0.57, 0.06)	0.113	181	− 0.20	(− 0.50, 0.11)	0.211
Repetitive sit-to-stand (FY *z*-score)	194	− 0.01	(− 0.37, 0.35)	0.963	170	0.00	(− 0.36, 0.35)	0.995	187	0.26	(− 0.08, 0.59)	0.129	167	0.27	(− 0.11, 0.65)	0.158
Physical performance score (FY *z*-score)	202	− 0.38	(− 0.77, 0.00)	0.050	177	− 0.34	(− 0.71, 0.03)	0.072	198	− 0.27	(− 0.64, 0.09)	0.143	174	− 0.22	(− 0.59, 0.16)	0.260
	*N*	Odds ratio	95% CI	*p* value	*N*	Odds ratio	95% CI	*p* value	*N*	Odds ratio	95% CI	*p* value	*N*	Odds ratio	95% CI	*p* value
Tinetti balance test < 10 s	212	1.79	(0.84, 3.81)	0.129	188	1.40	(0.60, 3.28)	0.440	213	1.50	(0.75, 3.01)	0.251	188	1.50	(0.62, 3.61)	0.366
Low physical performance score	202	2.01	(0.96, 4.23)	0.065	177	2.46	(0.98, 6.15)	0.055	198	1.67	(0.77, 3.64)	0.196	174	1.38	(0.54, 3.53)	0.496

^a^Adjusted for age, BMI, social class, smoker status, alcohol consumption, physical activity, dietary calcium intake and years since menopause and HRT use in women

Cognitive impairment on the Strawbridge questionnaire was associated with poorer physical performance score in men but not in women in the unadjusted analysis. In women, cognitive impairment on the Strawbridge questionnaire was associated with a weaker grip strength; however, statistical significant was lost when confounders were adjusted for.

Table 3 shows the associations between MMSE score (and MMSE score < 24) and physical performance outcomes in men and women.

**Table 3:** MMSE scores and MMSE score < 24 as explanatory variables for physical performance outcomes

	Men								Women							
	Unadjusted				Adjusted^a^				Unadjusted				Adjusted^a^			
MMSE score																
	*N*	Regression coefficient	95% CI	*p* value	*N*	Regression coefficient	95% CI	*p* value	*N*	Regression coefficient	95% CI	*p* value	*N*	Regression coefficient	95% CI	*p* value
Grip (kg)	221	0.32	(− 0.05, 0.69)	0.091	193	0.04	(− 0.33, 0.41)	0.827	221	0.00	(− 0.29, 0.29)	0.998	196	− 0.11	(− 0.45, 0.23)	0.517
Gait speed (FY *z*-score)	207	0.04	(− 0.01, 0.09)	0.112	180	0.03	(− 0.02, 0.08)	0.224	205	0.08	(0.02, 0.13)	0.004	181	0.06	(0.01, 0.11)	0.017
Repetitive sit-to-stand (FY *z*-score)	196	− 0.04	(− 0.10, 0.01)	0.120	172	− 0.04	(− 0.09, 0.01)	0.155	187	− 0.01	(− 0.07, 0.05)	0.746	167	− 0.01	(− 0.07, 0.06)	0.793
Physical performance score (FY *z*-score)	204	0.06	(0.00, 0.12)	0.049	179	0.05	(− 0.00, 0.11)	0.055	198	0.10	(0.05, 0.16)	< 0.001	174	0.07	(0.01, 0.13)	0.032
	*N*	Odds ratio	95% CI	*p* value	*N*	Odds ratio	95% CI	*p* value	*N*	Odds ratio	95% CI	*p* value	*N*	Odds ratio	95% CI	*p* value
Tinetti balance test < 10 s	214	0.94	(0.83, 1.06)	0.307	190	0.93	(0.82, 1.07)	0.320	213	0.87	(0.78, 0.97)	0.013	188	0.93	(0.81, 1.08)	0.353
Low physical performance score	204	0.89	(0.79, 1.00)	0.046	179	0.88	(0.77, 1.01)	0.079	198	0.83	(0.72, 0.96)	0.009	174	0.84	(0.71, 0.99)	0.042
MMSE score < 24																
	*N*	Regression coefficient	95% CI	*p* value	*N*	Regression coefficient	95% CI	*p* value	*N*	Regression coefficient	95% CI	*p* value	*N*	Regression coefficient	95% CI	*p* value
Grip (kg)	221	− 2.66	(− 6.22, 0.89)	0.141	193	0.91	(− 2.74, 4.56)	0.624	221	− 0.61	(− 3.06, 1.84)	0.626	196	0.27	(− 2.28, 2.83)	0.834
Gait speed (FY 7-score)	207	− 0.90	(− 1.40, − 0.39)	0.001	180	− 0.73	(− 1.27, − 0.20)	0.007	205	− 0.34	(− 0.76, 0.08)	0.110	181	− 0.30	(− 0.69, 0.09)	0.128
Repetitive sit-to-stand (FY 7-score)	196	0.82	(0.29, 1.34)	0.002	172	0.53	(− 0.03, 1.09)	0.065	187	0.05	(− 0.42, 0.52)	0.822	167	0.11	(− 0.40, 0.62)	0.667
Physical performance score (FY 2-score)	204	− 1.07	(− 1.62, − 0.52)	< 0.001	179	− 0.82	(− 1.38, − 0.26)	0.004	198	− 0.54	(− 1.03, − 0.06)	0.029	174	− 0.42	(− 0.89, 0.06)	0.087
	*N*	Odds ratio	95% CI	*p* value	*N*	Odds ratio	95% CI	*p* value	*N*	Odds ratio	95% CI	*p* value	*N*	Odds ratio	95% CI	*p* value
Tinetti balance test < 10 s	214	3.13	(1.12, 8.78)	0.030	190	2.33	(0.67, 8.07)	0.183	213	2.06	(0.87, 4.89)	0.103	188	1.42	(0.50, 4.02)	0.507
Low physical performance score^b^	204	12.92	(1.67, 99.81)	0.014					198	2.06	(0.67, 6.36)	0.207	174	1.91	(0.48, 7.56)	0.354

^a^Adjusted for age, BMI, social class, smoker status, alcohol consumption, physical activity, dietary calcium intake and years since menopause and HRT use in women

^b^Numbers were very low for the MMSE < 24 vs. low physical performance score in men and were insufficient to run the adjusted model

In women, MMSE score was associated with gait speed, physical performance score and higher prevalence of low physical performance score (≤ 9) following adjustments for confounders. As in men, MMSE score was associated with physical performance score. However, following adjustment for confounders, this association became non-significant. In men, MMSE score < 24 was associated with poorer gait speed, repetitive sit-to-stand, modified Tinetti balance test and physical performance score. In men, MMSE < 24 was strongly associated with the risk of low physical performance (OR 12.9, 95% CI 1.67, 99.8, *p* = 0.01), although we were unable to run adjusted models due to low numbers.

### Associations between cognitive impairment on bone parameters

Bone mineral content (BMC) and bone mineral density (BMD) were higher in men than in women at both hip and spine (data not shown). Male mean radial (4 and 66% slices) and tibial (4, 14, 38 and 66% slices) bone parameters as assessed by pQCT and HRpQCT scans were significantly greater than those in females (*p* < 0.001). Few statistically significant relationships were found between DXA, pQCT or HRpQCT outcomes and either cognitive frailty, as assessed by the Strawbridge questionnaire, or MMSE score in both men and women (unadjusted and adjusted, data not shown).

## Discussion

In this study, we utilised the Strawbridge cognitive domain and MMSE to investigate the potential association between cognitive impairment and both physical performance status, and bone mineral density and architecture in a community-dwelling cohort of elderly adults. We have shown MCI is associated with poor physical performance, but not bone density or microarchitecture in this cohort. It is recognised that physical performance decline is one of the most important predictors of future adverse health consequences such as disability, institutionalisation, hospitalisation and need for home healthcare assistance [24]. Thus, given the findings of this study, and the magnitude and speed of population ageing, identification of individuals with MCI is an important health priority.

Decreasing physical activity (PA) or maintaining a persistent physically inactive lifestyle is associated with greater decline in physical performance [25]. Furthermore, studies have demonstrated regular PA is a protective factor against cognitive impairment in the elderly, particularly women [26, 27]. Severely cognitively impaired individuals demonstrate significant reductions in levels of habitual physical activity [28]. It is possible that cognition-related behaviour changes may exacerbate age-related bone loss. In this study, we have demonstrated that MCI is associated with poorer physical performance. This decline in physical performance may lead to reduction in habitual physical activity and potentially lead to immobility, resulting in BMD loss over time. This process is likely to be exacerbated as further cognition-related behaviours develop as the degree of cognitive decline increases.

In this study, we demonstrated no association between MCI and bone mineral density, size or microarchitecture at the hip, radius or tibia. However, a previous study [29] has demonstrated that bone mineral architecture is reduced in individuals with severe cognitive impairment and Alzheimer’s disease (AD). These results suggest that BMD loss might be directly proportional to the degree of cognitive impairment. A possible explanation is that cognitive-related neurodegenerative brain changes may directly affect central control of bone remodelling. Atrophy of the limbic system, including the hypothalamus, increases with the degree of cognitive impairment and is a prominent hallmark of AD [30]. The hypothalamus regulates homeostatic metabolic processes, including the major role of regulating bone mass. Therefore, we may be able to account for our findings by proposing that individuals with MCI do not possess the amount of atrophy within the hypothalamus to affect regulation of bone mass, thus preserving their BMD. However, as further neurodegeneration occurs within the limbic system, the imbalance in bone remodelling homeostasis is impaired resulting in greater bone turnover and bone mineral loss, as seen in AD. Further studies assessing brain atrophy, including the limbic system, to examine the hypothesis that neurodegeneration may contribute to loss of bone mass are required.

Longitudinal studies of the relationship between cognitive function and physical performance in subsets of patients have suggested a link. In 177 UK patients with moderate to severe cognitive impairment (MMSE 11–23) followed up for a 1-year period, many neuropsychological, physical and functional performance measures declined [31]. Baseline gait speed was significantly associated with decline in verbal function, while controlling for age, education, dementia drug use and baseline cognitive performance [31]. In other works, in a US-randomised controlled trial of daily engagement of meaningful activity in adults of mean age 71 years with mild cognitive impairment, change in self-rated performance predicted change in depressive symptoms, suggesting that targeting physical performance in this group is worthwhile [32]. In another randomised controlled trial of cognitive and physical activity in 308 older Japanese adults with mild cognitive impairment [33], compared with the control group, the combined activity group showed significantly greater scores on the MMSE. Finally, malnutrition must be considered as a possible contributing factor in the development of sarcopenia or presarcopenia in adults with MCI; this can be targeted in interventions as appropriate [34]. These findings suggest not only a link between MCI and physical performance, but that intervention is feasible and beneficial in a range of populations.

Much of the available literature reporting associations between cognitive impairment and physical performance have been performed in Asian populations [35–40]. Decreased physical performance, as assessed by the short performance battery test, was associated with subsequent cognitive decline in 298 subjects of mean age 72 years at baseline [35] and a high prevalence of sarcopenia (> 20%) was recognised in Japanese subjects attending a memory loss clinic [36]. Reduced muscle strength and function was demonstrated in a Taiwanese population where the prevalence of sarcopenia was 7% [37] while sarcopenia was significantly associated with cognitive impairment and depressive symptoms among otherwise healthy older men living in a Taiwanese veterans retirement community [38]. While in another study that considered relationships between metabolic and atherosclerotic disease with outcomes in a Chinese population, cognitive impairment, high waist-hip ratio, diabetes, stroke and heart disease were all independently associated with higher physical frailty with adjustment for age, physical activity level and appendicular skeletal muscle mass [39]. In a final study of Chinese older adults > 65 years, the cognitively impaired group had weaker grip strength and performed worse in the physical function tests [40]. In rare European data, among some 1600 UK men enrolled in the British Regional Heart Study, compared with participants in the normal cognitive ageing group, participants with significant cognitive impairment were more likely to have a BMI > 30 and to be sarcopenic [41].

One of the limitations of this study is the lack of a standardised diagnostic tool for MCI. Therefore, by using the Strawbridge questionnaire and MMSE, we may have under-/overestimated MCI in our cohort. Additionally, our analyses are cross-sectional, and longitudinal data are now needed. Finally, appreciation that the Strawbridge questionnaire provides a subjective measure of cognitive impairment must be made. By contrast, one of the study’s strengths is the study population. The individuals recruited were a non-selective community-dwelling population who were born in Hertfordshire and continued to live there at the age of 60–75 years. HCS characteristic and mortality patterns have previously been demonstrated to be similar to those of England [42], thus generalisation to the wider population of older adults can be made. Using standardised batteries for physical performance tests and bone mineral architecture analysis which have well-known predictive ability for adverse health outcomes in older adults was another strength.

Longitudinal studies objectively assessing the full spectrum of cognitive impairment within a community-dwelling population would be beneficial to confirm our results. Additionally, it may identify which stage of cognitive impairment is associated with increased falls and reduction in bone mineral architecture.

## Conclusions

This study suggests mild cognitive impairment in elderly community-dwelling adults is associated with poor physical performance especially in men but not altered bone density or microarchitecture.

## References

[1] Lopez OL, Kuller LH, Becker JT, Dulberg C, Sweet RA, Gach HM, DeKosky ST (2007). Incidence of dementia in mild cognitive impairment in the cardiovascular health study cognition study. Arch Neurol.

[2] Tschanz JT, Welsh-Bohmer KA, Lyketsos CG, Corcoran C, Green RC, Hayden K, Norton MC, Zandi PP, Toone L, West NA, Breitner JCS, the Cache County Investigators (2006). Conversion to dementia from mild cognitive disorder—the Cache County study. Neurology.

[3] Burns JM, Cronk BB, Anderson HS, Donnelly JE, Thomas GP, Harsha A, Brooks WM, Swerdlow RH (2008). Cardiorespiratory fitness and brain atrophy in early Alzheimer disease. Neurology.

[4] Camarda R, Camarda C, Monastero R, Grimaldi S, Camarda LK, Pipia C, Caltagirone C, Gangitano V (2007). Movements executions in amnestic mild cognitive impairment and Alzheimer’s disease. Behav Neurol.

[5] Kluger A, Gianutsos JG, Golomb J, Ferris SH, Reisberg B (1997). Motor/psychomotor dysfunction in normal aging, mild cognitive decline, and early Alzheimer’s disease: diagnostic and differential features. Int J Psychogeriatr.

[6] Barrett-Connor E, Edelstein S, Corey-Bloom J, Wiederholt W (1998). Weight loss precedes dementia in community-dwelling older adults. J Nutr Health Aging.

[7] Douglas A, Letts L, Richardson J (2011). A systematic review of accidental injury for older adults with and without dementia. Arch Gerontol Geriatr.

[8] Sato Y, Honda Y, Hayashida N, Iwamoto J, Kanoko T, Satoh K (2005). Vitamin K deficiency and osteopenia in elderly women with Alzheimer’s disease. Arch Phys Med Rehabil.

[9] Dudek M, Pietraszkiewicz F, Drozdzowska B (2008). Alzheimer’s disease and osteoporosis: common risk factors or one condition predisposing to the other?. J Orthopaedics, Trauma Rehabil.

[10] Harlein J, Halfen RJG, Dassen T, Lahmann NA (2011). Falls in older hospital inpatients and the effects of cognitive impairment: a secondary analysis of prevalence studies. J Clin Nurs.

[11] van Doorn C, Gruber-Baldini AL, Zimmerman S, Hebel JR, Port CL, Baumgarten M, Quinn CC, Taler G, May C, Magaziner J; Epidemiology of Dementia in Nursing Homes Research Group. Dementia as a risk factor for falls and fall injuries among nursing home residents. J Am Geriatr Soc. 2003 Sep;51(9):1213–8 10.1046/j.1532-5415.2003.51404.x 12919232

[12] Rudolph JL, Zanon NM, Jones RN, Marcantionio ER, Fong TG, Yang FM (2010). Hospitalisation in community-dwelling persons with Alzheimer’s: frequency and causes. J Am Geriatr Soc.

[13] Dennison EM, Syddall HE, Aihie Sayer A, Gilbody HJ, Cooper C (2005). Birth weight and weight at 1 year are independent determinants of bone mass in the seventh decade: the Hertfordshire cohort study. Paediatr Res.

[14] Folstein M, Folstein S, McHugh P (1975). Mini-mental state. J Psychol Res.

[15] Strawbridge WJ, Shema SJ, Balfour JL, Higby HR, Kaplan GA (1998). Antecedents of frailty over three decades in an older cohort. J Gerontol Series B, Psychol Sci Soc Sci.

[16] Bohannon RW (1986). Test-retest reliability of hand held dynamometry during a single session of strength assessment. Phys Ther.

[17] Posiadlo D, Richardson S (1991). The timed “up and go”: a test of basic functional mobility for frail elderly persons. J Am Geriatr Soc.

[18] Csuka M, McCarty DJ (1985). Simple method for measurement of lower extremity muscle strength. Am J Med.

[19] Tinetti ME (1986). Performance oriented assessment of mobility problems in the elderly patient. Am Geiatr Soc.

[20] Guralnik JM, Simonsick EM, Ferrucci L, Glynn RJ, Berkman LF, Blazer DG (1994). A short physical performance battery assessing lower extremity function: association with self-reported disability and prediction of mortality and nursing home admission. J Gerontol.

[21] Boutroy S, Bouxsein ML, Munoz F, Delmas PD (2005). In vivo assessment of trabecular bone microarchitecture by high-resolution peripheral quantitative computed tomography. J Clin Endocrinol Metab.

[22] Laib A, Hauselmann HJ, Ruegsegger P (1998). In vivo high resolution 3D-QCT of the human forearm. Technol Health Care.

[23] Burghardt AJ, Buie HR, Laib A, Majumdar S, Boyd SK (2010). Reproducibility of direct quantitative measures of cortical bone microarchitecture of the distal radius and tibia by HRpQCT. Bone.

[24] Guralnik JM, Simonsick EM, Ferrucci L (1995). Lower extremity function in persons over the age of 70 years as a predictor of subsequent disability. N Engl J Med.

[25] Stenholm S, Koster A, Valkeinen H (2016). Association of physical activity history with physical function and mortality in old age. J Am Geriatr Soc.

[26] Pareja-Galeno H, Garatachea N, Lucia A (2015). Exercise as a polypill for chronic diseases. Program Mol Biol Transl Sci.

[27] de Labra C, Guimaraes-Pinheio C, Maseda A, Lorenzo T, Millan-Calenti JL (2015). Effects of physical exercise interventions in frail older adults: a systematic review of randomised controlled trials. BMC Geriatr.

[28] Ensrud KE, Lui LY, Paudel ML, Schousboe JT, Kats AM, Cauley JA, McCulloch CE, Yaffe K, Cathon PM, Hillier TA, Taylor BC (2017). Effects of mobility and cognition on hospitalisation and inpatient days in women in late life. J Gerontol.

[29] Loskutova N, Honea RA, Vidoni E, Brooks WM, Burns JM (2009). Bone density and brain atrophy in early Alzheimer’s disease. J Alzheimers Dis.

[30] Whitwell JL, Petersen RC, Negash S, Weigand SD, Kantarci K, Ivnik RJ, Knopman DS, Boeve BF, Smith GE, Jack CR (2007). Patterns of atrophy differ among specific subtypes of mild cognitive impairment. Arch Neurol.

[31] Taylor ME, Lasschuit DA, Lord SR, Delbaere K, Kurrle SE, Mikolaizak AS, Kvelde T, Close JCT (2017). Slow gait speed is associated with executive function decline in older people with mild to moderate dementia: a one year longitudinal study. Arch Gerontol Geriatr.

[32] Ellis JL, Altenburger P, Lu Y (2017) Change in depression, confidence, and physical function among older adults with mild cognitive impairment. J Geriatr Phys Ther:1 10.1519/JPT.0000000000000143 PMC5910300 29059120

[33] Shimada H, Makizako H, Doi T, Park H, Tsutsumimoto K, Verghese J, Suzuki T (2017) Effects of combined physical and cognitive exercises on cognition and mobility in patients with mild cognitive impairment: a randomized clinical trial. J Am Med Dir Assoc 10.1016/j.jamda.2017.09.019 29153754

[34] Tay L, Leung BP, Wee S, Tay KS, Ali N, Chan M, Lim WS (2017). Association of nutrition and immune-endocrine dysfunction with muscle mass and performance in cognitively impaired older adults. Arch Gerontol Geriatr.

[35] Moon JH, Moon JH, Kim KM, Choi SH, Lim S, Park KS, Kim KW, Jang HC (2016). Sarcopenia as a predictor of future cognitive impairment in older adults. J Nutr Health Aging.

[36] Sugimoto T, Ono R, Murata S, Saji N, Matsui Y, Niida S, Toba K, Sakurai T (2016). Prevalence and associated factors of sarcopenia in elderly subjects with amnestic mild cognitive impairment or Alzheimer disease. Curr Alzheimer Res.

[37] Huang CY, Hwang AC, Liu LK, Lee WJ, Chen LY, Peng LN, Lin MH, Chen LK (2016). Association of dynapenia, sarcopenia, and cognitive impairment among community-dwelling older Taiwanese. Rejuvenation Res.

[38] Hsu YH, Liang CK, Chou MY, Liao MC, Lin YT, Chen LK, Lo YK (2014). Association of cognitive impairment, depressive symptoms and sarcopenia among healthy older men in the veterans retirement community in southern Taiwan: a cross-sectional study. Geriatr Gerontol Int.

[39] Lee JS, Auyeung TW, Leung J, Kwok T, Leung PC, Woo J (2011). Physical frailty in older adults is associated with metabolic and atherosclerotic risk factors and cognitive impairment independent of muscle mass. J Nutr Health Aging.

[40] Auyeung TW, Kwok T, Lee J, Leung PC, Leung J, Woo J (2008). Functional decline in cognitive impairment—the relationship between physical and cognitive function. Neuroepidemiology.

[41] Papachristou E, Ramsay SE, Lennon LT, Papacosta O, Iliffe S, Whincup PH, Wannamethee SG (2015). The relationships between body composition characteristics and cognitive functioning in a population-based sample of older British men. BMC Geriatr.

[42] Dennison EM, Syddall HE, Aihie Sayer A, Gilbody HJ, Cooper C (2005). Birth weight, infant weight gain and cause-specific mortality: the Hertfordshire cohort study. Am J Epidemiol.

